# Knowledge and Practice Behaviours of Obstetricians/Gynecologists and Midwives Concerning Periodontal Health and Pregnancy

**DOI:** 10.3290/j.ohpd.b4586823

**Published:** 2023-11-02

**Authors:** Camille Bechina, Guillaume Bonvillain, Gildas Rethore, Assem Soueidan, Norbert Winer, Yoann Maitre, Xavier Struillou

**Affiliations:** a Assistant, Department of Periodontology, Faculty of Dental Surgery, Nantes Université, Nantes. Idea, study design, data collection, data interpretation, wrote the manuscript.; b Postgraduate Student, Department of Periodontology, Faculty of Dental Surgery, Nantes Université, Nantes. Study design, data collection.; c Associate Professor with Anatomical Sciences Department, CHU Nantes, Nantes Université, UIC Odontology 11, Nantes, France. Contributed to critical proofreading and discussion, as read and approved the manuscript.; d Professor of Periodontology, CHU Nantes, Nantes Université, Periodontology Department, UIC Odontology 11, Nantes, France; Nantes Université, CHU Nantes, INSERM, Regenerative Medicine and Skeleton, RMeS, Nantes, France. Project supervisor, study design, synthesis of results, read and approved the manuscript.; e Professor and Head of the Department of Medical Gynecology and Obstetrics, CHU Nantes, Nantes Université, Department of Gynecology and Obstetrics, Nantes, France. Contributed to critical proofreading and discussion, read and approved the manuscript.; f Associate Professor, Department of Prevention, Epidemiology, Health Economics, Forensic-Odontology Law, UIC Odontology 11, Nantes, France. Study design and statistical analysis.; g Associate Professor of Periodontology, CHU Nantes, Nantes Université, Periodontology Department, UIC Odontology 11, Nantes, France; Nantes Université, CHU Nantes, INSERM, Regenerative Medicine and Skeleton, RMeS, Nantes, France. Idea, study design, synthesis of results, read and approved the manuscript.

**Keywords:** attitude, health knowledge, midwife, obstetrician/gynecologist, practice, periodontal disease, pregnancy

## Abstract

**Purpose:**

The purpose of the present study was to evaluate the level of knowledge of prenatal health professionals concerning the relationship between periodontal diseases and pregnancy complications, as well as their professional implications in the oral health field.

**Materials and Methods:**

A questionnaire was distributed to obstetricians/gynecologists, interns specialised in obstetrics/gynecology, midwives, and student midwives at Loire Atlantique and Vendée hospitals (France). The questionnaire included 5 sociodemographic questions and 14 questions regarding the level of knowledge about the relationship between periodontal diseases and pregnancy complications as well as the professionals’ level of involvement in oral health care.

**Results:**

Twenty-three obstetricians/gynecologists and 55 midwives responded to the questionnaire. Preterm delivery and chorioamnionitis were the most frequently mentioned complications of pregnancy, whereas the risk of pre-eclampsia was rarely mentioned. Half of the professionals said they were aware of the oral manifestations of pregnancy. Gingivitis and an increased risk of caries were the most frequently mentioned items, whereas epulis was the least frequently mentioned item. The level of involvement of prenatal care practitioners in oral health care was limited due to a lack of competence and time. Nevertheless, 64% of the participants discussed the risks of poor oral hygiene with their patients.

**Conclusion:**

There is good knowledge among French gynecologists/obstetricians and midwives regarding the oral manifestations of pregnancy. However, there is still a lack of knowledge concerning the links between periodontal diseases and pregnancy complications. The involvement and behaviour of pregnancy professionals in the oral health field is inadequate. The present survey highlights the need to improve the initial and continuing education of obstetricians and midwives on this topic.

Periodontal diseases are inflammatory diseases of microbial origin that result in the progressive destruction of all the components of the periodontium (epithelium, connective tissue, ligaments and alveolar bone). They can be defined as multifactorial infectious diseases. Periodontal diseases are characterised by clinical symptoms and signs that may include gingival inflammation, bleeding on probing, attachment loss, alveolar bone loss, and tooth loss.^19,25^

The presence of periodontitis represents a major source of bacteria and inflammatory mediators that can enter the bloodstream and reach the placenta and amniotic fluid, thus affecting the course of pregnancy.^23,33,37^

The association between periodontal diseases and pregnancy complications has been known for more than two decades, although the exact aetiology is not fully understood.^2,28^ The complications of pregnancy resulting from this combination are preterm delivery, low birth weight, and pre-eclampsia.^6,9,14,24,27^

In France, approximately 50% of the adult population (35–64 years old) presents with periodontal disease and at least one site with severe attachment loss (> 6 mm).^4^ In the pregnant population, the prevalence of periodontitis varies between 12.1% and 37.7%.^16^

As is apparent from numerous randomised studies, there are differing opinions concerning the effectiveness of periodontal disease treatment in reducing adverse pregnancy outcomes^7,15,18,29,36^ and the optimal time to treat periodontal disease to prevent pregnancy complications.

Considering the prevalence of periodontal disease, the range of adverse pregnancy outcomes that has been associated with periodontal disease, and the unclear treatment efficacy, it is important to promote the prevention of periodontal disease before and during pregnancy.

During pregnancy, women encounter several health professionals, such as general practitioners, obstetricians/gynecologists, midwives, and dentists. In France, the National Agency for Accreditation and Evaluation in Health (ANAES) has set up a system that allows any pregnant woman to have a free consultation for a complete check-up with an oral health professional and, if needed, free care from the 4th month of pregnancy until 12 days after delivery.^1^

Among 904 women following childbirth, the MaterniDent study, which was performed in France in 2013, showed that 56% had not consulted a dentist during their pregnancy, 26% had gone for a pre-existing problem, and only 18% had gone for a preventive oral check-up.^34^

Therefore, more active involvement of prenatal care practitioners, including obstetricians/gynecologists and midwives, is important to increase pregnant women’s awareness of periodontal disease and pregnancy complications.

The aim of the present study was to evaluate the level of knowledge concerning the relationship between periodontal disease and pregnancy complications among health professionals involved in pregnancy, including obstetricians/gynecologists and midwives.

## Materials and Methods

### Study Design and Location

A cross-sectional study was conducted from January 2021 to February 2022 to assess the level of knowledge of the association between periodontal disease and pregnancy complications in the maternity departments of the university hospitals of Loire Atlantique, Nantes (CHU) and Vendée, La Roche sur Yon (CHD). The present study was approved by the Nantes Group of Ethics in Health.

### Study Population

The study population comprised health professionals supervising the management of pregnancy, including obstetricians/gynecologists and midwives (students or graduates).

A total of 78 health professionals (n = 23 obstetricians/gynecologists and interns specialised in obstetrics/gynecology; n = 55 midwives and student midwives) participated in the present study.

The study population was selected according to the inclusion and exclusion criteria shown in Table 1.

**Table 1:** Inclusion and exclusion criteria

Inclusion criteria	Exclusion criteria
– Graduate midwives, working in the hospital, agreeing to participate in the study – Student at the Nantes midwifery school agreeing to participate in the study and being in the fourth of fifth year of study – Graduated obstetricians/gynecologists, working in the hospital, agreeing to participate in the study – Interns specialised in obstetrics/gynecology, working in the hospital, agreeing to participate in the study.	– Refusal to participate in the study – Student not following his/her course at the Nantes school of midwives – Student in the second or third year of studies – Midwife not practicing in a hospital center – Doctors and interns not specialised in obstetrics/gynecology – Doctors and interns not practicing in a hospital center

### Experimental Design – Evaluation of Criteria

The main objective was to evaluate the level of knowledge regarding the relationship between periodontal health and pregnancy complications among midwives and obstetricians/gynecologists in the maternity departments of the university hospitals of Loire Atlantique and Vendée.

Two anonymous, digital questionnaires were distributed to obstetricians/gynecologists and midwives by email. The questionnaires consisted of 5 sociodemographic items and 14 items aimed at collecting data on the level of knowledge regarding the association between periodontal disease and pregnancy complications.

The degree of professional involvement of obstetricians/gynecologists and midwives in the maintenance of oral health and the prevention of oral health diseases in their patients was also recorded.

To assess the level of knowledge, the questions were multiple choice. To determine the professional degree of involvement, the questions were graded.

### Statistical Analysis

All of the data were entered into a database using Microsoft Excel 2019. The results are presented as numbers/percentages, means/standard deviations, or medians/interquartile ranges. Statistical analyses were performed using R software package 4.1.3 (2022-03-10).

The following statistical tests were used: the Shapiro-Wilk normality test, Wilcoxon-Mann‒Whitney test, Fisher’s exact test, Χ^2^ test of independence, Spearman’s rank correlation, and the Kruskal-Wallis test.

## Results

### Knowledge of Prenatal Care Practitioners

Approximately 140 questionnaires were e-mailed to the practitioners in the maternity departments of the university hospitals of Loire Atlantique, Nantes (CHU) and Vendée, La Roche sur Yon (CHD).

A total of 78 health care professionals (n = 23 obstetricians/gynecologists and interns specialising in obstetrics and gynecology; n = 55 midwives and student midwives) answered the questionnaire and took part in the present study. Of these professionals, 45% were students or interns, and 55% were graduates. The average age of the practitioners was 31.94 years, and the majority of the population was female (95%).

**Fig 1:**
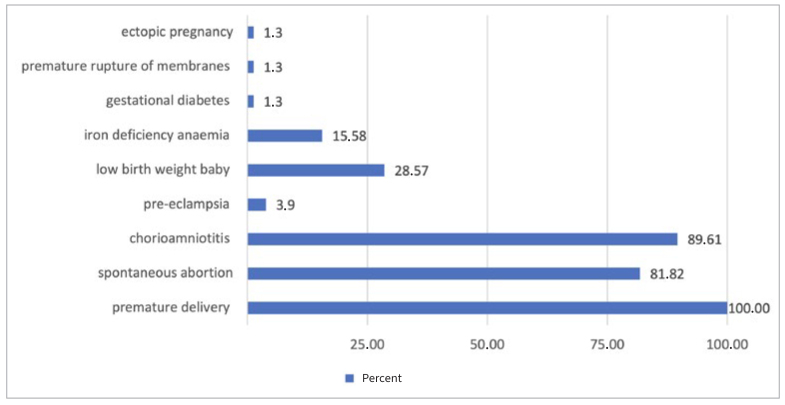
Adverse pregnancy outcomes reported by prenatal-care practitioners as a consequence of periodontal diseases.

**Fig 2:**
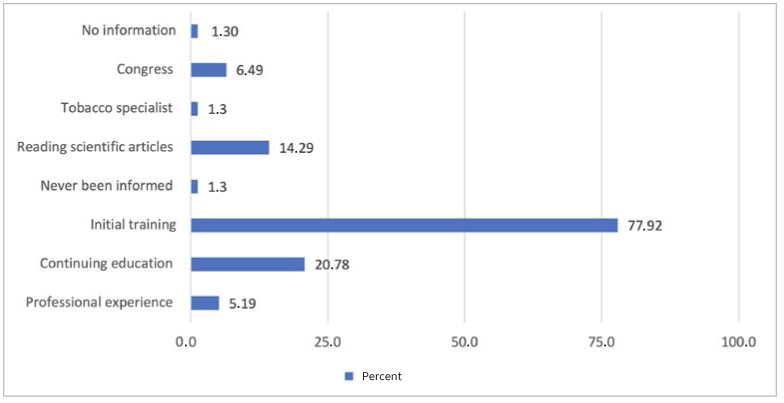
Means by which prenatal-care practitioners received knowledge regarding the association between periodontal disease and pregnancy complications.

**Fig 3:**
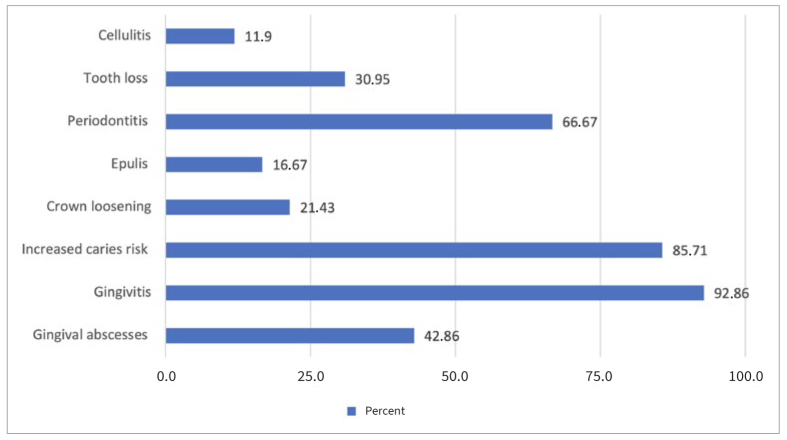
Oral manifestations associated with pregnancy complications reported by prenatal-care practitioners.

Figures 1 to 3 show the knowledge of prenatal care practitioners concerning periodontal diseases, adverse pregnancy outcomes, oral manifestations, and the channels through which they obtained this knowledge.

Regarding the impact of periodontal diseases on pregnancy complications, 99% of the participants responded that they were aware of this issue. The most adverse pregnancy outcome mentioned as a consequence of periodontal diseases was preterm delivery (100%), followed by chorioamnionitis (89.61%) and spontaneous abortion (81.82%).

The most cited means of accessing knowledge was initial training (77.92%), followed by continuing education (20.78%).

54% of professionals reported having knowledge of oral manifestations resulting from pregnancy. The most cited oral manifestation was gingivitis (92.86%), followed by increased caries risk (85.71%) and periodontitis (66.67%). There was a statistically significant difference according to profession in the reported knowledge of the effects of pregnancy on the oral cavity. Midwives reported a higher level of knowledge of these consequences than did obstetricians/gynecologists (Table 2).

**Table 2:** Reported knowledge of the effects of pregnancy on the oral cavity by occupation

Profession	No, N = 36	Yes, N = 42	p <0.001*
Obstetricians/gynecologists	18 (50%)	5 (12%)	
Midwives	18 (50%)	37 (88%)	

*Χ^2^ test for independence.

### Involvement of Prenatal-Care Practitioners in Oral Health

On average, the prenatal-care practitioners reported consulting with a pregnant woman 4.26 (±2.78) times regarding her pregnancy.

In addition, 42% of prenatal-care practitioners knew the recommended period for an oral check-up (2nd and 3rd trimesters) (Table 3).

**Table 3:** Reported knowledge regarding the recommended period of consultation with a dentist during pregnancy

	%	N = 78
**In France, a visit to the dentist during pregnancy is recommended…**		
In the first trimester	36	28
In the second trimester	32	25
In the third trimester	10	8
I have no idea	22	17

Among the prenatal-care practitioners, 64% discussed the risks of poor oral hygiene with their patients. There was a statistically significant difference between having or starting a discussion and not having/starting a discussion about the risks of poor oral hygiene and the number of visits (p < 0.001). There was also a statistically significant correlation between the number of visits and frequency of holding discussions (p = 0.003).

Among the prenatal-care practitioners, 94% reported that they did not perform oral examinations. If an examination was performed, they reported redness, bleeding, and suppuration as the most common findings. For those who reported that they did not perform oral examinations, a lack of competence (79.45%) and a lack of time (34.25%) were the most cited causes.

Only 41% of the prenatal-care practitioners gave oral hygiene advice during consultations, and 47% verified whether pregnant women underwent oral health check-ups during pregnancy. Follow-up regarding the completion of an oral health check-up statistically significantly differed according to the number of check-ups during pregnancy (p = 0.028), which indicated that a higher number of visits favoured its implementation.

**Fig 4:**
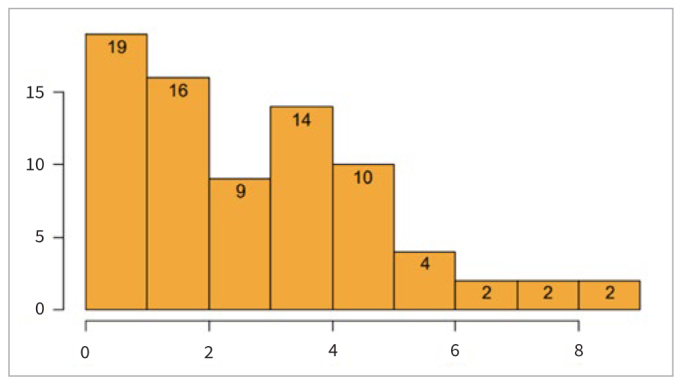
Number of staff according to prenatal-care practitioners’ level of involvement scores (x-axis: points scored; y-axis: staff).

A score for the involvement of prenatal-care practitioners in the oral health of pregnant women was established using the four questions listed in Table 4. The responses ranged from 0 to 3 (0 = no; 1 = yes, sometimes; 2 = yes, often if I think the patient has risk factors; and 3 = yes, systematically), with the minimum score being 0 and the maximum score being 12 (Table 5 and Fig 4).

**Table 4:** Degree of involvement of pregnancy professionals in the oral health care of pregnant women

	%	n = 78
**Do you discuss with your patients the potential risks associated with poor oral hygiene during pregnancy?**		
Yes, systematically	7,7	6
Yes, often	15	12
Yes, sometimes	41	32
No	36	28
**Do you perform a clinical examination of the oral cavity of your patients?**		
Yes, systematically	0	0
Yes, often	0	0
Yes, sometimes	6,4	5
No	94	73
**Do you give oral hygiene advice to your patients?**		
Yes, systematically	1,3	1
Yes, often	9	7
Yes, sometimes	31	24
No	59	46
**Do you check that they carry out a visit to their dentist?**		
Yes, systematically	6,4	5
Yes, if I think the patient has risk factors	17	13
Yes, sometimes	24	19
No	53	41

**Table 5:** Involvement score results

Average (standard deviation)	Minimum	1st Quantile	Median	3rd Quantile	Maximum
2.31 ± 2.15	0.00	1.00	2.00	3.37	9.00

## Discussion

For many years, studies have shown an association between periodontal disease and pregnancy complications.^24^ Despite this, studies conducted in France and around the world have shown a certain general level of acquired knowledge but a lower level of involvement of prenatal care practitioners in the oral health domain.^10,13,35^

In France, epidemiological studies on this subject are still limited.^6,13^ Previous studies have focused on practicing prenatal professionals (obstetricians/gynecologists and midwives) and dentists, and the samples ranged from 87 to 100 prenatal care practitioners. The study objectives were similar, as they investigated the knowledge and involvement of prenatal care practitioners in the oral health field.

The majority of studies published in the literature involve a limited number of practitioners, namely, between 55 and 190.^10,35^ According to other studies on the same subject, the number of participants in the present study was average. In contrast to the other studies, the present study also included students, which provided a more varied population.^6,10^

The first part of the study focused on the level of knowledge of prenatal-care practitioners concerning the link between periodontal disease and pregnancy complications. The present results were in agreement with previous studies.^10,13,35^

Previous studies have shown that oral infections, periodontitis in particular, lead to a risk of complications during pregnancy, such as pre-eclampsia, premature delivery, and low birth weight.^11,23^ The risk of preterm birth due to oral infections was known to the entire study population.^9,20,22^ The notion of low-birth-weight babies was only mentioned by less than a third of the study population, and the notion of pre-eclampsia was barely mentioned. However, many studies have shown an association between pre-eclampsia and periodontitis.^30,32^ Pregnancy professionals, whether teachers or students, underestimate the risk of pre-eclampsia, and there is a lack of information on this topic among pregnancy professionals.

The vast majority of responders cited a risk of chorioamnionitis and spontaneous abortion due to oral infections. However, there is a paucity of literature on this, and hypotheses regarding the association of periodontal diseases with pregnancy complications have not been firmly established.^3,8^ Because infections can contaminate the fetoplacental unit and cause pregnancy complications, it is likely that the pregnancy professionals considered infection in a broad sense. However, the pregnancy professionals were not familiar with oral health issues.

Previous studies have reported that pregnancy induces a change in the immune system of pregnant women. Pregnancy hormones, such as progesterone, enhance the growth of periodontopathogenic bacteria, such as *Porphyromonas gingivalis*. This decrease in immunity associated with a modification of the bacterial flora and oral fluids explains the appearance of gingival/periodontal disease in pregnant women.^12,26,33,37^ The notion of gingival infection, including gingivitis and periodontitis, was mostly cited by obstetricians/gynecologists and midwives. This may have been the result of a bias because the subject of the study was known by the participants answering the questionnaire.

The increased risk of caries was also cited. Dietary habits and gastroesophageal reflux in early pregnancy can weaken the enamel, leading to caries if combined with poor oral hygiene. Studies have shown that during pregnancy, salivary factors change in a manner which increases the risk of developing caries.^17,38^ Pregnancy-induced epulis was only rarely mentioned by prenatal-care practitioners. There is a misunderstanding regarding crown loosening, which is not a pregnancy-related oral complication.^31^ The knowledge acquired may have been based on the gynecological field and not the oral field, and the lack of information among both working professionals and students suggested that there were gaps in this interdisciplinary knowledge in the initial training.

The second part of the present study evaluated the level of involvement of prenatal-care practitioners in the oral health care of their patients. The established score (Fig 4) suggested a low level of involvement of prenatal-care practitioners in the oral field. Only 8% of the study population received at least the average score (0–12 points).

Regarding the questions asked to determine the involvement score, more than half of the practitioners mentioned discussing poor oral hygiene with their patients during consultations. In contrast, there was little involvement in terms of giving oral hygiene advice or performing oral examinations during consultations. Among the professionals who reported that they performed oral examinations, periodontal disease was the main feature sought. Because the survey referred to periodontal disease, there may have been a bias in reporting which oral manifestation was sought. Pregnant women were only rarely examined for caries, which may have been due to the lack of knowledge of oral pathologies among health professionals. These results agreed with the indication of the lack of competence in the oral health field in prenatal-care practitioners who do not perform oral cavity examinations.^21^

In France, an oral health consultation is provided for pregnant women from the 4th month of pregnancy until 12 days after delivery. In the present study, more than half of the prenatal care practitioners did not know when their patients should consult their dentists. The limited knowledge regarding this examination and a lack of verification suggested a lower level of involvement in oral health care.

The results of similar studies in France as well as elsewhere agreed with the present results.^6,10,13,35^ Over the last ten years, this subject has entered the scientific debate due to the increased awareness of prevention and interdisciplinary relations. However, the results remain unchanged, and there has been no progress. The level of knowledge of pregnancy professionals concerning periodontal diseases and complications of pregnancy as well as oral manifestations of pregnancy remains correct. However, clinical behaviour still does correspond to this level of knowledge. The involvement of prenatal-care practitioners in the oral health field remains limited and should be improved in the coming years.^6,10,13^

The first step to overcome this is to review multidisciplinary training in initial and continuing education. Better integration of the oral health field will enable better care for pregnant women and establish an interdisciplinary relationship between prenatal-care practitioners and dentists.

The lack of competence and time cited by practitioners in the present study may be addressed by placing posters and/or leaflets calling attention to the association between periodontal diseases and pregnancy complications in waiting rooms or handing them to individual patients. Thus, pregnant women, who are the chief interested parties, will have the necessary information on this subject. This will make it possible to include oral health issues in pregnancy care, and prenatal-care practitioners will be able to refer more patients to dentists.

The present study had several limitations. The present findings may have been biased, because the subject of the study was periodontal diseases, which may have influenced the professionals’ answers. In addition, the majority of the study population was female. Because these women may have had children, they may have been more aware of pregnancy complications.

## Conclusion

The present study evaluated the knowledge of obstetricians/gynecologists and midwives regarding the association between periodontal disease and pregnancy complications as well as the oral manifestations of pregnancy. Compared to similar studies, the necessary knowledge had not been fully acquired by prenatal-care practitioners.

The involvement and behaviour of pregnancy professionals in the oral health field is still inadquate. The present survey highlighted the need to improve the initial and continuing education of obstetricians and midwives on this topic. The oral health domain is still not included to a sufficient extent in the monitoring of pregnant women. Because pregnancy professionals are the primary caretakers of pregnant women, it is necessary to include information about gingival and periodontal health in their training. The prevention of periodontal disease remains the optimal strategy to prevent pregnancy complications.

To optimise the care of pregnant women, an interdisciplinary relationship between prenatal-care practitioners and dentists is necessary. The introduction of an information form or checklist on gingival health for pregnant women would allow better involvement of prenatal-care practitioners in the oral field. In the long term, the establishment of good clinical practice guidelines would be the ideal means of ensuring that all health professionals are aware of this topic.

## Supplementary Material

Questionnaire for the assessing the oral health training of registered midwives and obstetricians/gynecologists in France

About you:

1. You are:
  □ A man
  □ A woman
2. How old are you? .........years old

Question for midwives:

3. Are you:
  □ Student midwife (specify year of study): ........................
    (go directly to question 5)
  □ A graduate midwife in a public health facility
  □ Midwife graduated from a private health care institution
4. In what year did you graduate as a midwife? .................
5. In which city are you studying/have you studied? .........................

Question for obstetricians/gynecologists:

3. What is your profession?
  □ Gynecologist-obstetrician: ...............
    (Specify if hospital practitioner, associate professor, head of department...)
  □ Intern (specify internship semester): ..........................................
    (go directly to question 6)
  □ Other: ...................................................
4. What year did you graduate? .....................
5. In which city did you obtain your degree? .....................................

Pregnancy and oral hygiene:

6. In your opinion, can the periodontal health of a pregnant woman affect the health of the newborn and the smooth progress of the pregnancy?
  □ No (go directly to question 9)
  □ Yes
  □ Don’t know (go directly to question 9)
7. If yes, what are the risks to pregnancy of poor periodontal health? (Several answers possible)
  □ Pre-eclampsia
  □ Spontaneous abortion
  □ Chorioamniotitis
  □ Delivery haemorrhage
  □ Premature delivery
  □ Low weight baby
  □ Gestational diabetes
  □ Ectopic pregnancy
  □ Iron-deficiency anaemia
  □ Other: .................................
8. How were you informed about this?
  □ Initial training
  □ Continuing education
  □ Congresses
  □ Reading scientific articles
  □ Media
  □ Other: ........................
9. Are you aware of the effects of pregnancy on the oral domain?
  □ Yes
  □ No (go directly to question 11)
10. In your opinion, what are they?
  □ Increased risk of caries
  □ Gingival abscesses
  □ Cellulitis
  □ Crown loosening
  □ Gingivitis
  □ Epulis (benign tumour growth of the gums)
  □ Periodontitis
  □ Tooth loss
  □ Other: .....................................

Maintaining the oral health of pregnant women as part of your professional practice:

11. On average, how many times do you see a patient during her pregnancy? ..................... times
12. Do you discuss with your patients the potential risks associated with poor oral hygiene when monitoring pregnancy?
  □ Yes, systematically
  □ Yes, often
  □ Yes, sometimes
  □ No
13. Do you carry out a clinical examination of your patients’ oral cavity?
  □ No (go directly to question 15)
  □ Yes, sometimes
  □ Yes, often
  □ Yes, systematically
14. If yes, what are you looking for (multiple answers possible):
  □ Bleeding
  □ Suppuration
  □ Redness
  □ Restorations: crowns, amalgams, composites, etc.
  □ Cavities
  □ Other: ...........................................
15. If no, for what reason(s)?
  □ Lack of time
  □ Lack of equipment
  □ Lack of skills
  □ This exam does not seem relevant
  □ Other: ...........................................
16. In France, a consultation with a dentist during pregnancy:
  □ Is recommended during the first trimester
  □ Is recommended during the second trimester
  □ Is recommended during the third trimester
  □ I have no idea
17. Do you give oral hygiene advice to your patients?
  □ Yes, sometimes
  □ Yes, often
  □ Yes, systematically
  □ No
18. Do you check that they actually visit their dentist?
  □ Yes, systematically
  □ Yes, sometimes
  □ Yes, if I consider that the patient has risk factors
  □ No
